# Proteomic analysis reveals semaglutide impacts lipogenic protein expression in epididymal adipose tissue of obese mice

**DOI:** 10.3389/fendo.2023.1095432

**Published:** 2023-03-21

**Authors:** Ruiyi Zhu, Shuchun Chen

**Affiliations:** ^1^ Department of Internal Medical, Hebei Medical University, Shijiazhuang, Hebei, China; ^2^ Department of Internal Medical, Hebei General Hospital, Shijiazhuang, Hebei, China

**Keywords:** semaglutide, white adipose tissue, obesity, proteomics, lipid metabolism

## Abstract

**Background and objectives:**

Obesity is a global health problem with few pharmacologic options. Semaglutide is a glucagon-like peptide-1 (GLP-1) analogue that induces weight loss. Yet, the role of semaglutide in adipose tissue has not yet been examined. The following study investigated the mechanism of semaglutide on lipid metabolism by analyzing proteomics of epididymal white adipose tissue (eWAT) in obese mice.

**Methods:**

A total of 36 C57BL/6JC mice were randomly divided into a normal-chow diet group (NCD, n = 12), high-fat diet (HFD, n = 12), and HFD+semaglutide group (Sema, n = 12). Mice in the Sema group were intraperitoneally administered semaglutide, and the HFD group and the NCD group were intraperitoneally administered an equal volume of normal saline. Serum samples were collected to detect fasting blood glucose and blood lipids. The Intraperitoneal glucose tolerance test (IPGTT) was used to measure the blood glucose value at each time point and calculate the area under the glucose curve. Tandem Mass Tag (TMT) combined with liquid chromatography-tandem mass spectrometry (LC-MS/MS) were used to study the expression of eWAT, while cellular processes, biological processes, corresponding molecular functions, and related network molecular mechanisms were analyzed by bioinformatics.

**Results:**

Compared with the model group, the semaglutide-treated mice presented 640 differentially expressed proteins (DEPs), including 292 up-regulated and 348 down-regulated proteins. Bioinformatics analysis showed a reduction of CD36, FABP5, ACSL, ACOX3, PLIN2, ANGPTL4, LPL, MGLL, AQP7, and PDK4 involved in the lipid metabolism in the Sema group accompanied by a decrease in visceral fat accumulation, blood lipids, and improvement in glucose intolerance.

**Conclusion:**

Semaglutide can effectively reduce visceral fat and blood lipids and improve glucose metabolism in obese mice. Semaglutide treatment might have beneficial effects on adipose tissues through the regulation of lipid uptake, lipid storage, and lipolysis in white adipose tissue.

## 1 Introduction

Obesity is a multifactorial chronic disease characterized by excessive fat accumulation in adipose tissue, which can lead to insulin resistance, hypertension, and dyslipidemia (1). Dyslipidemia is an important link between obesity and the development of type 2 diabetes mellitus(T2DM), cardiovascular disease (CVD), and certain types of cancer, such as breast cancer and colon adenomas (2). Semaglutide, a glucagon-like peptide-1 (GLP-1) analogue, has been reported to induce weight loss among overweight or obese adults, as well as to have a beneficial effect on cardiometabolic health in these populations (3). Gabery et al. reported that semaglutide lowers rodent body weight *via* distributed neural pathways (4). Moreover, Pontes-da-Silva et al. discovered that semaglutide reduces insulin resistance, liver inflammation, and endoplasmic reticulum stress in obese mice (5). Another study reported that semaglutide has beneficial effects on a pro-inflammatory pathway, PDX1, and PPAR-alpha and gamma, by reducing the lesion on the islet. However, the impact of semaglutide on weight loss was found to have little relevance in the pancreatic islet caused by insulin resistance (6).

There are evidences shows GLP-1 analogues directly signal to adipose tissue. Previous study provided evidence for the presence of GLP-1 receptor in adipose tissue (7). The beneficial effects of GLP-1 have been found to be associated with changes in the adipogenesis, lipolysis, thermogenesis and anti-inflammation process. A recent study discovered that GLP-1 down-regulated the expression of adipogenic/lipogenic genes on adipose tissue *in vivo* and *in vitro*, while increasing that of lipolytic markers and adiponectin (8). Zhang proved that GLP-1 analogue liraglutide decreased adipocyte size, increased secretion of FGF21, and promoted phosphorylation of LKB1, AMPK and Acetyl coenzyme A carboxylase 1 (ACC1) in white adipose tissue of DM mice (9). Similarly, Shao also found that with liraglutide treatment, visceral adipose tissue of mice was reduced with AMPK activation and Akt suppression, which was associated with reduction of lipogenetic process (10). GLP-1 analogues may also target epicardial adipose tissue GLP-1R and therefore reduce local adipogenesis, improve fat utilization and induce brown fat differentiation (11). Moreover, Wan showed that GLP-1 analogue supaglutide reduces HFD-induced obesity, which is associated with increased Ucp-1 in white adipose tissue of mice (12). Absalon discovered the anti-diabetic effects of liraglutide was mediated by transient upregulation of IL-6, which activates canonical IL-6R signaling resulted in adipose tissue browning and thermogenesis linked with STAT3 activation (13). GLP-1 also has anti-inflammatory effects on adipose tissue, it reduces macrophage infiltration and directly inhibits inflammatory pathways in adipocytes and adipose tissue macrophages, possibly contributing to the improvement of insulin sensitivity (14). Administration of GLP-1 analogues exenatide or liraglutide reduced inflammatory marker mRNA in adipose tissue of T2DM obese subjects (8). However, no study has examined the role of semaglutide on adipose tissue so far.

In mammals, the white adipose tissue (WAT) is the major organ that stores extra energy from diets in the form of triglycerides (TG) or fat, which can be mobilized to meet energy demands (15). Therefore, dysfunction in white adipose tissue metabolism is a cardinal event in the development of insulin resistance and metabolic disorders (16). In this study, we investigated changes in lipid metabolism proteomes in epididymal white adipose tissue (eWAT) of diet-induced obese (DIO) mice in response to semaglutide intervention by TMT combined with LC-MS/MS to provide greater insight into the mechanism of lipid metabolism by semaglutide.

## 2 Methods

### 2.1 Mice

A total of 36 male C57BL/6JC mice (7-week-old, 16−20 g) purchased from Hebei INVIVO Laboratory Animal Technology Co., Ltd. (Hebei, China) were housed (3–5 mice per cage) in a pathogen-free facility in a temperature-controlled room (22°C) with a 12-h light/dark cycle, and were given free access to food and water. All animal studies (including the mice euthanasia procedure) were done in compliance with the regulations and guidelines of Hebei General Hospital institutional animal care and conducted according to the AAALAC and the IACUC guidelines.

The current study only investigated male mice since they are more susceptible to diet-induced obesity and diet-induced insulin resistance than female mice (17). After one week of acclimatization, mice were randomly distributed into two groups and fed with either a normal-chow diet (NCD, n = 12) or a high-fat diet (60% fat, 20% carbohydrate, 20% protein, total calories 524kcal/100 g) (n = 24). After 12 weeks of feeding, the high-fat diet group was further divided into the HFD+saline group (HFD, n = 12) and HFD+semaglutide group (Sema, n = 12). Mice in the Sema group were intraperitoneally administered semaglutide (30nmol/kg/d, Novo Nordisk, Bagsværd, Denmark), whereas the NCD and HFD groups were treated with saline. Body weight were measured once a week.

After 12 weeks of treatment, glucose tolerance tests and metabolic measurements were carried out. Mice were fasted for 12 h prior to sacrifice. At the end of the experiment, mice were anesthetized with 1% sodium pentobarbital (60 mg/kg) intraperitoneal injection. Blood was collected from the retro-orbital sinus and placed into sterile tubes containing 1 mm EDTA, after which the mice were euthanized. Interscapular BAT (iBAT) and epididymal WAT (eWAT) were collected, weighted, and subjected to hematoxylin and eosin (H&E) staining or snap-frozen in liquid nitrogen and stored at 80°C until analysis.

### 2.2 Glucose tolerance tests and AUC measurements

Blood glucose was monitored by examining tail vein blood using the Roche blood glucose monitoring system. The intraperitoneal glucose tolerance test (i.p. GTT) was carried out after 12 weeks of sumaglutide treatment. For i.p. GTT, mice were fasted overnight and were given 2 g of 50% glucose/kg body weight *via* intraperitoneal injection. Tail vein blood glucose levels were measured at 0, 15, 30, 60, 90, and 120 min, and the area under the curve (AUC) was obtained.

### 2.3 Serum analysis

Serum samples were separated by centrifugation at 4°C and stored at -80°C until used for measurements. Insulin levels were detected by enzyme-linked immunosorbent assay (ELISA) using the Mouse INS (Insulin) ELISA Kit (Elabscience Biotechnology Co., Ltd). Triglyceride (TG), total cholesterol (TC), LDL-C, and HDL-C assay kits were purchased from Jiancheng Biology Institution PeproTech (Nanjing, China). The above assays were conducted according to the manufacturers’ instructions.

### 2.4 Histopathological analysis

Both sides of epididymal white adipose tissues and interscapular brown adipose tissues were removed, weighed, fixed in 4% paraformaldehyde for 48 h, and immersed in the dehydration box for dehydration and wax leaching. The wax-soaked tissues were embedded. The paraffin blocks were cut into 4 µm. After de-paraffinization, they were stained with hematoxylin and eosin (H&E). The target area of the tissue was selected for 200x imaging using an Eclipse Ci-L photomicroscope (Nikon Eclipse E100), and the tissue was imaged to fill the entire field of view as much as possible to ensure consistent background light in each photograph. After imaging was completed, Image-Pro Plus 6.0 (Nikon DS-U3) analysis software was used to uniformly measure 5 muscle fiber diameters in each section with mm as the standard unit; the number of adipocytes was counted in 3 fields of view in each section and the total area of adipocytes was measured in the field of view as well as the area of the field of view; the average area of adipocytes was calculated as = total area of adipocytes/number of adipocytes, and the adipose cell density was calculated as = number of adipocytes/area of field of view.

### 2.5 Protein digestion and peptide labelling

**Figure 1:**
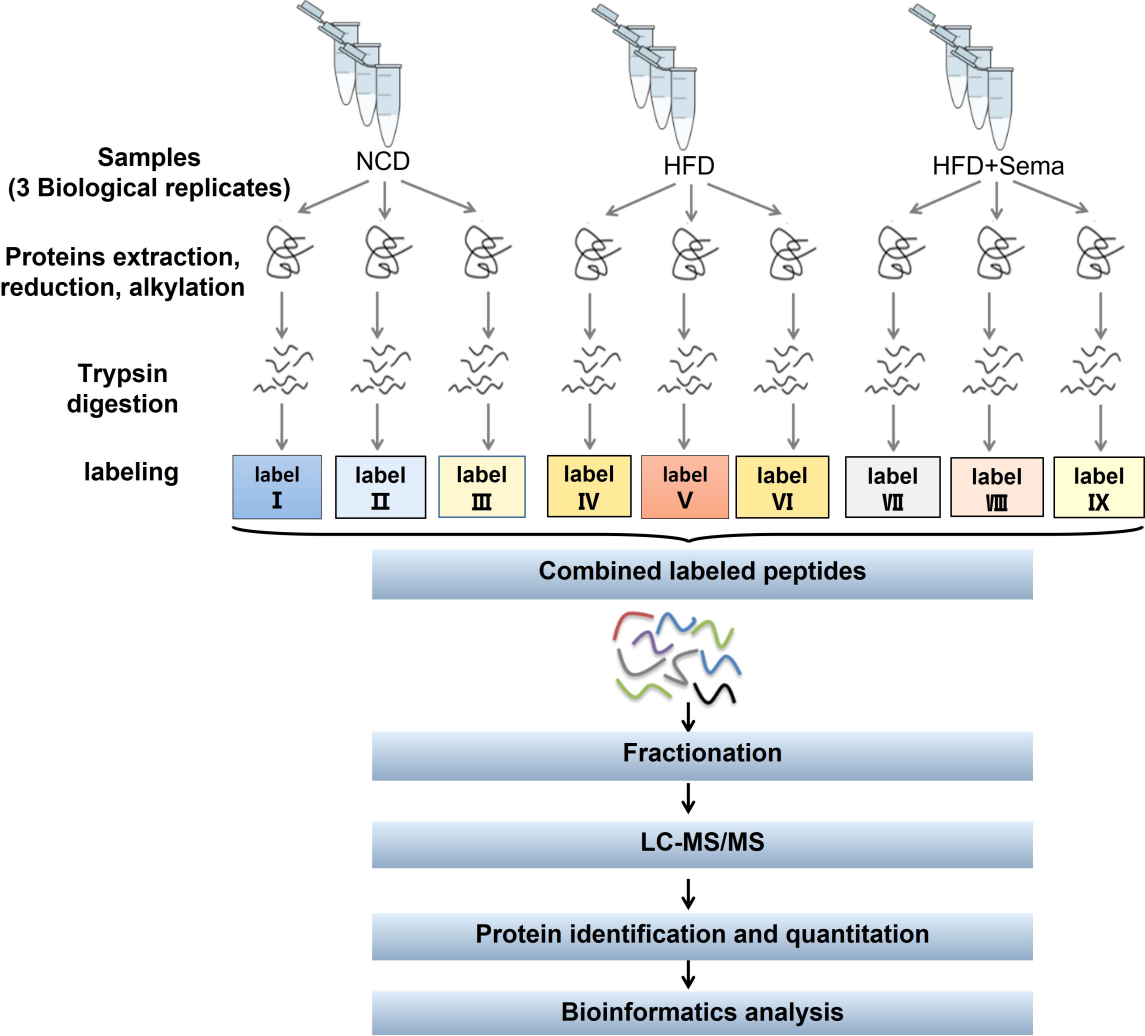
Study workflow. eWAT samples in each group (≥ 600 mg per group) were subjected to three biological replicates (each replicate was a mixture of eWAT from 5–7 mice). LC-MS/MS, liquid chromatography-tandem mass spectrometry; GO, gene ontology; KEGG, Kyoto Encyclopedia of Genes and Genomes.

The flowchart of proteomics and bioinformatics analysis is shown in **Figure 1**. Nine epididymal white adipose tissues (3 tissues/group) were ground by liquid nitrogen into cell powder, lysed, and extracted in SDT (4%SDS, 100mM Tris-HCl, 1mM DTT, pH7.6) buffer. The amount of protein was quantified with the BCA Protein Assay Kit (Bio-Rad, USA). Protein digestion by trypsin was performed according to the filter-aided sample preparation (FASP) procedure described by Matthias Mann (18). The digest peptides of each sample were desalted on C18 Cartridges (Empore™ SPE Cartridges C18 (standard density), concentrated by vacuum centrifugation, and reconstituted in 40 µl of 0.1% (v/v) formic acid. The purity of proteins was determined by sodium dodecyl sulfate-polyacrylamide gel electrophoresis (SDS-PAGE) system, after which 100 μg peptide mixture of each sample was labeled using iTRAQ reagent (Applied Biosystems)/TMT reagent (Thermo Scientific) according to the manufacturer’s instructions.

### 2.6 LC-MS/MS analysis

Labeled peptides were fractionated by High pH Reversed-Phase Peptide Fractionation Kit (Thermo Scientific). The collected fractions were desalted on C18 Cartridges and concentrated by vacuum centrifugation. LC-MS/MS analysis was performed on a Q Exactive mass spectrometer (Thermo Scientific) that was coupled to Easy nLC (Thermo Fisher Scientific) for 60/90 min. The peptides were loaded onto a reverse phase trap column (Thermo Scientific Acclaim PepMap100, 100 μm*2 cm, nanoViper C18) connected to the C18-reversed-phase analytical column (Thermo Scientific Easy Column) in buffer A (0.1% Formic acid) and separated with a linear gradient of buffer B (84% acetonitrile and 0.1% Formic acid) at a flow rate of 300 nl/min controlled by IntelliFlow technology. The MS raw data for each sample were searched using the MASCOT engine (Matrix Science, London, UK; version 2.2) embedded into Proteome Discoverer 1.4 software for identification and quantitation analysis.

### 2.7 Bioinformatics analysis

Cluster 3.0 (http://bonsai.hgc.jp/~mdehoon/software/cluster/software.htm) and Java Treeview software (http://jtreeview.sourceforge.net) were used to perform hierarchical clustering analysis. CELLO (http://cello.life.nctu.edu.tw/), a multi-class SVM classification system, was used to predict protein subcellular localization. Protein sequences were searched using the InterProScan software to identify protein domain signatures from the InterPro member database Pfam. The protein sequences of the selected differentially expressed proteins were locally searched using the NCBI BLAST+ client software (ncbi-blast-2.2.28+-win32.exe) and InterProScan to find homologue sequences, after which gene ontology (GO) terms were mapped, and sequences were annotated using the software program Blast2GO. Following annotation steps, the studied proteins were blasted against the online Kyoto Encyclopedia of Genes and Genomes (KEGG) database (http://geneontology.org/) to retrieve their KEGG orthology identifications, after which they were mapped to pathways in KEGG. Enrichment analysis was applied based on Fisher’ exact test, considering the whole quantified proteins as the background dataset. Benjamini- Hochberg correction for multiple testing was further applied to adjust derived p-values. Functional categories and pathways with p-values < 0.05 were considered statistically significant.

### 2.8 Statistical analysis

All mice experiments were analyzed using Prism 8 GraphPad Software (San Diego, California). All data were analyzed using ordinary or repeated-measures one-way or two-way ANOVA; when indicated, Tukey’s or Dunnett’s were used for multiple comparison tests. All data are expressed as mean ± SEM (Standard error of mean). Differences with a *p-*value of < 0.05 were considered statistically significant.

## 3 Results

### 3.1 Semaglutide reduces body weight and improves metabolic profiles

**Figure 2:**
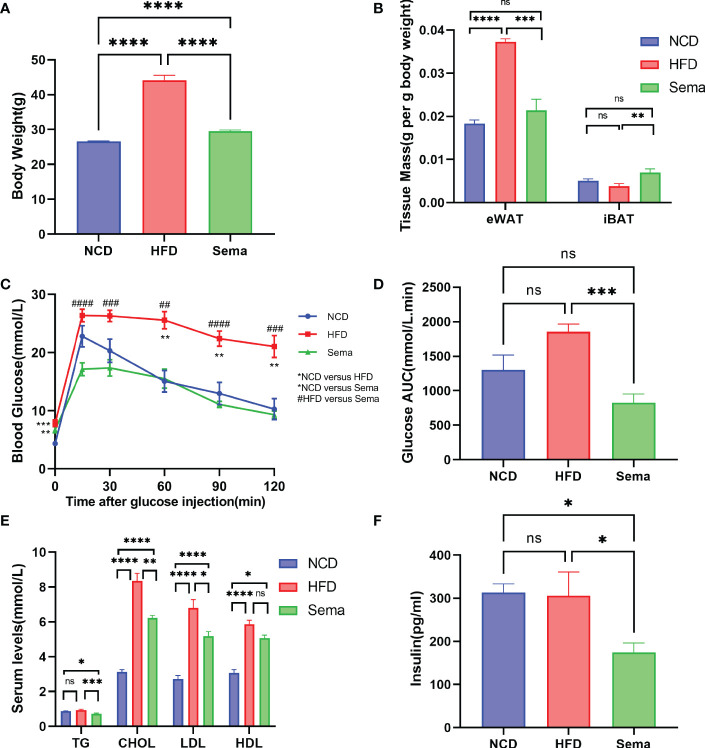
The effects of semaglutide on reducing body weight and visceral obesity and improving metabolic profiles. **(A)** Average body weight of each group before being sacrificed (n = 8). **(B)** Fat mass/body weight ratio (n = 8). **(C, D)** i.p. GTT after semaglutide treatment and area of the curve (AOC) generated by the area under the curve (AUC), with subtraction of the basal glucose (n = 8). **(E, F)** Determination of serum TC, TG, LDL-C, HDL-C, and insulin concentration (n =8); Data were presented as mean ± SEM. “ns” p > 0.05, *p < 0.05, **p < 0.01, ***p < 0.001, ****p < 0.0001, ^#^p < 0.05, ^##^p < 0.01, ^###^p < 0.001, ^####^p < 0.0001. NCD, normal control; HFD, high-fat diet-induced mice; Sema, HFD diet treated by semaglutide.

At the end of the treatment, the body weight of mice in the Sema group was significantly lower than that of the HFD group mice (**Figure 2A**), and the eWAT weight/body weight ratio showed differences consistent with body weight, while the iBAT weight/body weight ratio was significantly higher (p<0.05) in Sema group (**Figure 2B**), suggesting that semaglutide led to a weight-loss effect by lowering the proportion of visceral fat mass and increasing the brown fat mass relative to total body mass.

As shown in **Figures 2C, D**, there were no significant differences in fasting blood glucose levels between the Sema and HFD groups (*P* > 0.05). However, compared with the HFD group, semaglutide significantly reduced its blood glucose concentration at 15 min, 30 min, 60 min, 90 min, and 120 min, whilst the area under the blood glucose curve significantly decreased (*P* < 0.001). The fluctuation of blood glucose levels in HFD-fed mice was alleviated by semaglutide treatment, suggesting that semaglutide could improve the rate of glucose clearance and insulin sensitivity.

Next, we measured the content of TC, TG, LDL-C, HDL-C, and insulin (**Figures 2E, F**) in the serum obtained on the day the mice were sacrificed. TC, LDL-C, and HDL-C levels were significantly elevated in HFD-fed mice compared with NCD-fed mice, while the elevation of TC and LDL was significantly suppressed (p<0.05) after semaglutide treatment. In addition, the insulin and TG levels were comparable between HFD group mice and the control group but were significantly decreased with semaglutide treatment. However, no significant difference in HDL-C level was observed between HFD and Sema groups.

### 3.2 Semaglutide decreases the size of adipocytes in eWAT and iBAT

**Figure 3:**
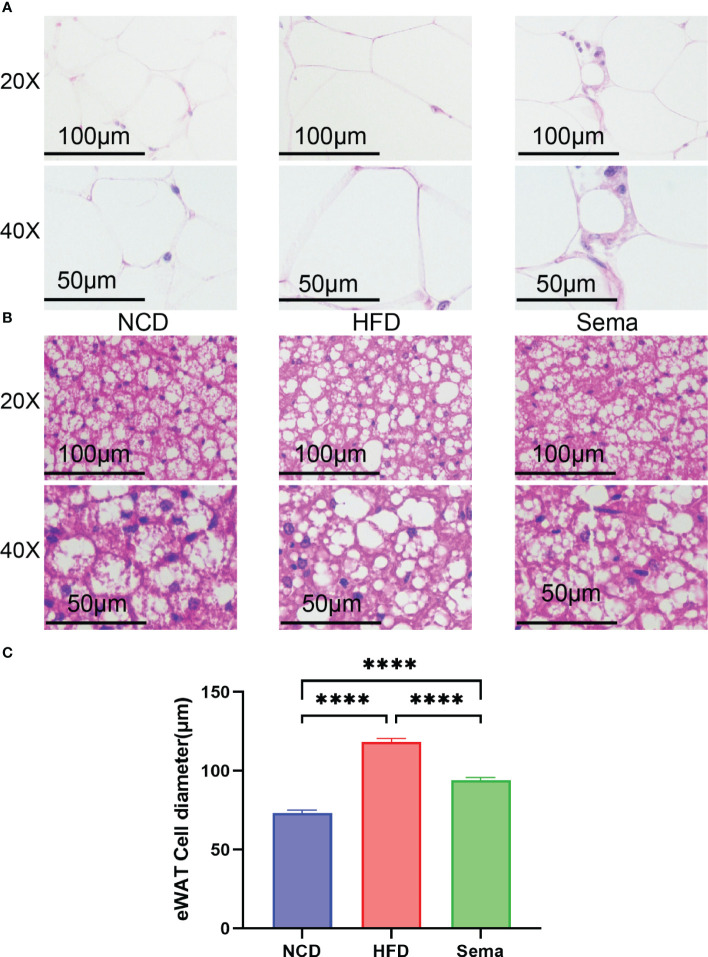
Representative image of histological changes of eWAT and iBAT. The size of adipocyte evaluated by staining with H&E. **(A)** Representative H&E staining from eWAT of mice. Scale bar, 100 µm, and 50 µm. (n = 3). **(B)** Representative H&E staining from iBAT of mice. Scale bar, 100 µm, and 50 µm. (n = 3). **(C)** The fat diameter of the adipocyte was quantified by Image **J** (n = 5). Statistical results are presented as the mean ± SEM. **** p < 0.0001 is an indicative significance compared with vehicle-treated right-side fat as self-control.

H&E staining showed that the adipocytes in eWAT of NCD group were similar in size and regular in shape, same patterns were observed in iBAT. Yet, compared with NCD group, the adipocytes in eWAT and iBAT of HFD group mice were significantly larger, with distinct morphologies and more observable in lipid droplets, which could be alleviated by the treatment of semaglutide (**Figures 3A, B**). Also, the diameter of adipocytes in eWAT was markedly decreased in Sema group, compared with HFD group (**Figure 3C**).

### 3.3 TMT-based quantitative proteomics analysis of eWAT

Quantitative proteomic analysis, based on TMT labeling, was conducted on the eWAT of NCD, HFD, and Sema groups (n = 3 mice per each group). A total of 53,911 peptide fragments were used, of which 48,914 were unique peptides corresponding to a total of 7590 proteins (**Figure S1**, **Table S1**). A satisfactory quality deviation was obtained during the data acquisition process using a high-quality Q Exactive mass spectrometer. The mass deviations of all the identified peptides were primarily distributed within 10 ppm, indicating that the identification results were accurate and reliable (**Figure S2A**). A great score with a median of 29.19 was attained, and more than 68.52% of peptides scored higher than 20 when evaluating each MS2 spectrogram (**Figure S2B**). The protein ratio distribution in the Sema/HFD groups is shown in **Figure S2C**. A 1.2-fold change cut-off, with P < 0.05, was used to indicate significant changes in the abundance of the DEPs in the Sema/HFD groups (**Table S2**).

### 3.4 The identification of differentially expressed proteins

**Figure 4:**
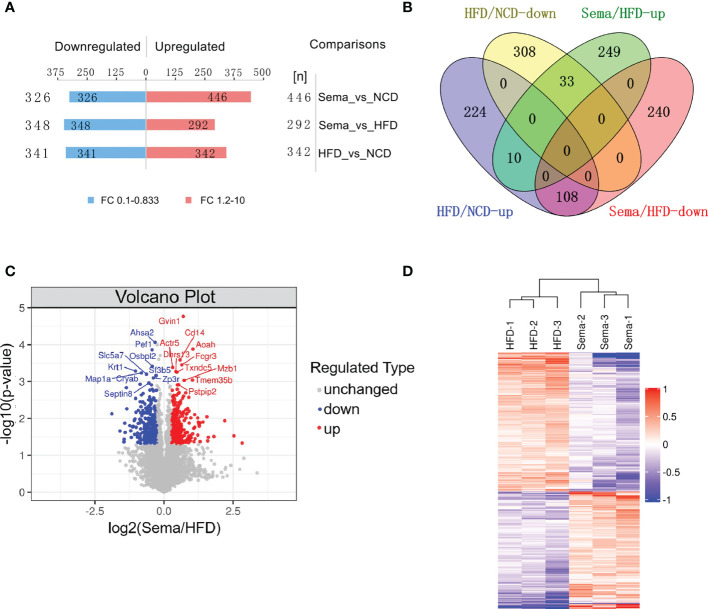
Identification and quantitative analysis of proteins. **(A)** The number of DEPs among each group. **(B)** The number of up-regulated and down-regulated proteins was compared between the two groups in Venn diagram. **(C)** Volcano plots show the distribution of significance and fold change of identified proteins between the Sema and HFD groups. The blue spots and red spots indicate significantly down-regulated and up-regulated proteins. Red indicates upregulation and blue represents downregulation. **(D)** Hierarchical clustering of DEPs from the Sema group compared with the HFD group. Red indicates upregulation, and blue represents downregulation.

A total of 683 DEPs, 342 up-regulated and 341 down-regulated, were identified in the HFD group versus the NCD group. Semaglutide treatment resulted in 640 DEPs, 292 up-regulated and 348 down-regulated, compared with the HFD group, and semaglutide induced 772 DEPs, 446 up-regulated and 326 down-regulated, compared with the NCD group (n = 3 per each group) (**Figure 4A**). Fold Changes ratios > 1.2 or < 0.83 and P-values (T-test) < 0.05 were considered to be DEPs. The list of the up- and down-regulated proteins between the Sema and HFD groups is shown in **Tables S3** and **S4**. In addition, a volcano plot and K-means clustering heatmaps were used to show the distribution of significance and fold change of the DEPs between the Sema and HFD groups (**Figures 4C, D**). As shown in **Table 1**, several proteins among the top 10 up-regulated DEPs were involved in the lipopolysaccharide catabolic process, cellular response to diacyl bacterial lipopeptide, antibody-dependent cellular cytotoxicity, apoptotic cell clearance, positive regulation of cell proliferation, cell migration including AOAH, CD14, FCGR3, TXND5, MZB1, and PPIP2. The top 10 down-regulated proteins participated in the ER to Golgi vesicle-mediated transport and cholesterol transport, and were the integral component of the membrane. There were 141 overlapping DEPs between Sema/HFD and HFD/NCD in Venn diagram, including 33 down-regulated and 108 up-regulated proteins with HFD reversed by Sema. (**Figure 4B**). The list of the DEPs reversed by semaglutide between HFD/NCD and Sema/HFD (**Table 2**).

**Table 1:** List of the top 10 DEPs significantly up/down-regulated between the Sema and HFD groups.

Accession	Protein Name	Gene Name	Sema / HFD
Q80SU7	Interferon-induced very large GTPase 1	Gvin1	1.626029763
O35298	Acyloxyacyl hydrolase	Aoah	2.069990226
P10810	Monocyte differentiation antigen CD14	Cd14	1.496559752
P08508	Low affinity immunoglobulin gamma Fc region receptor III	Fcgr3	1.566858006
Q80US4	Actin-related protein 5	Actr5	1.242135008
Q5SS80	Dehydrogenase/reductase SDR family member 13	Dhrs13	1.349912505
Q91W90	Thioredoxin domain-containing protein 5	Txndc5	1.381413158
Q3U0Y2	Transmembrane protein 35B	Tmem35b	2.04449729
Q9D8I1	Marginal zone B- and B1-cell-specific protein	Mzb1	1.658031446
Q99M15	Proline-serine-threonine phosphatase-interacting protein 2	Pstpip2	1.443331029
Q8N9S3	Activator of 90 kDa heat shock protein ATPase homolog 2	Ahsa2	0.801091655
P41242	Megakaryocyte-associated tyrosine-protein kinase	Matk	0.568717607
Q9DC63	F-box only protein 3	Fbxo3	0.722183075
P56375	Acylphosphatase-2	Acyp2	0.589639337
Q6TCG2	Membrane progesterone receptor epsilon	Paqr9	0.620337924
Q6IME9	Keratin, type II cytoskeletal 72	Krt72	0.562148059
B1AZA5	Transmembrane protein 245	Tmem245	0.633014045
Q80WR1	Tetraspanin-18	Tspan18	0.483043027
Q8BFY6	Peflin	Pef1	0.743245711
Q8BX94	Oxysterol-binding protein-related protein 2	Osbpl2	0.74768025

**Table 2:** List of the DEPs reversed by semaglutide between HFD/NCD and Sema/HFD.

Accession	Protein Name	Gene Name	Sema/HFD	HFD/NCD
O35855	Branched-chain-amino-acid aminotransferase, mitochondrial	Bcat2	1.281506424	0.687858846
O35930	Platelet glycoprotein Ib alpha chain	Gp1ba	1.558792701	0.76277469
O88986	2-amino-3-ketobutyrate coenzyme A ligase, mitochondrial	Gcat	1.282493527	0.675599541
P01631	Ig kappa chain V-II region 26-10		2.183937191	0.786278394
P04370	Myelin basic protein	Mbp	1.337736286	0.66124981
P07934	Phosphorylase b kinase gamma catalytic chain, skeletal muscle/heart isoform	Phkg1	1.810779455	0.635150402
P08074	Carbonyl reductase [NADPH] 2	Cbr2	1.422088101	0.795488833
P08553	Neurofilament medium polypeptide	Nefm	1.538117971	0.487675052
P24472	Glutathione S-transferase A4	Gsta4	1.554097442	0.50216426
P27573	Myelin protein P0	Mpz	1.73576827	0.507765755
P27931	Interleukin-1 receptor type 2	Il1r2	1.539524739	0.804402067
P51949	CDK-activating kinase assembly factor MAT1	Mnat1	1.231841048	0.717964191
P56565	Protein S100-A1	S100a1	1.556928633	0.560643756
Q05421	Cytochrome P450 2E1	Cyp2e1	1.481945121	0.400138198
Q3TC72	Fumarylacetoacetate hydrolase domain-containing protein 2A	Fahd2	1.346361794	0.653543367
Q3U0Y2	Transmembrane protein 35B	Tmem35b	2.04449729	0.708955297
Q5XJY4	Presenilins-associated rhomboid-like protein, mitochondrial	Parl	1.334386893	0.758964767
Q61024	Asparagine synthetase [glutamine-hydrolyzing]	Asns	1.318686424	0.710323152
Q61878	Bone marrow proteoglycan	Prg2	2.936173825	0.823299954
Q8BVZ5	Interleukin-33	Il33	1.346081593	0.737888785
Q8C0Q2	Zinc fingers and homeoboxes protein 3	Zhx3	1.223233196	0.698919446
Q8C7H1	Methylmalonic aciduria type A homolog, mitochondrial	Mmaa	1.266632979	0.738615031
Q8K0T0	Reticulon-1	Rtn1	1.439219824	0.768530397
Q8K3V7	Major intrinsically disordered Notch2-binding receptor 1	Minar1	1.485521325	0.432834834
Q8K4F5	Protein ABHD11	Abhd11	1.323545844	0.778982921
Q91WM2	Haloacid dehalogenase-like hydrolase domain-containing 5	Hdhd5	1.378265367	0.754543827
Q9CR59	Growth arrest and DNA damage-inducible proteins-interacting protein 1	Gadd45gip1	1.512518484	0.783963033
Q9CZ57	5-methylcytosine rRNA methyltransferase NSUN4	Nsun4	1.248652722	0.829931513
Q9D2R0	Acetoacetyl-CoA synthetase	Aacs	1.433225626	0.511798207
Q9D2V5	Protein AAR2 homolog	Aar2	1.466674803	0.71822847
Q9DCC4	Pyrroline-5-carboxylate reductase 3	Pycr3	1.245197813	0.792313915
Q9ERD7	Tubulin beta-3 chain	Tubb3	1.218067096	0.801430453
Q9Z211	Peroxisomal membrane protein 11A	Pex11a	1.401647745	0.555367229
A2AJ76	Hemicentin-2	Hmcn2	0.802742454	1.288415013
B1AZA5	Transmembrane protein 245	Tmem245	0.633014045	1.349864576
E9Q634	Unconventional myosin-Ie	Myo1e	0.62298073	1.804548698
E9Q6P5	Tetratricopeptide repeat protein 7B	Ttc7b	0.717785347	1.220191175
F7BWT7	Tetraspanin-15	Tspan15	0.647667159	1.36638246
O09131	Glutathione S-transferase omega-1	Gsto1	0.72555588	1.231584915
O09164	Extracellular superoxide dismutase [Cu-Zn]	Sod3	0.516482216	1.574537595
O35075	Vacuolar protein sorting-associated protein 26C	Vps26c	0.608732943	1.406154887
O35382	Exocyst complex component 4	Exoc4	0.824478971	1.220650116
O54998	Peptidyl-prolyl cis-trans isomerase FKBP7	Fkbp7	0.79323375	1.245320989
O55186	CD59A glycoprotein	Cd59a	0.39357848	1.585667381
O70318	Band 4.1-like protein 2	Epb41l2	0.689916081	1.248056386
O70325	Phospholipid hydroperoxide glutathione peroxidase	Gpx4	0.731072157	1.281299472
O70571	[Pyruvate dehydrogenase (acetyl-transferring)] kinase isozyme 4, mitochondrial	Pdk4	0.5890158	1.589726035
O88495	Melatonin-related receptor	Gpr50	0.592898971	1.576243268
O88822	Lathosterol oxidase	Sc5d	0.73134654	1.251946776
P00493	Hypoxanthine-guanine phosphoribosyltransferase	Hprt1	0.637009885	1.455198993
P02468	Laminin subunit gamma-1	Lamc1	0.626512243	1.213661846
P04925	Major prion protein	Prnp	0.708954669	1.245567387
P09541	Myosin light chain 4	Myl4	0.67612683	1.210510072
P10107	Annexin A1	Anxa1	0.455736333	2.177574731
P13020	Gelsolin	Gsn	0.768408546	1.318531459
P14069	Protein S100-A6	S100a6	0.724108013	1.470089004
P14824	Annexin A6	Anxa6	0.7645763	1.323836591
P16546	Spectrin alpha chain, non-erythrocytic 1	Sptan1	0.735131675	1.220197343
P20152	Vimentin	Vim	0.662676811	1.382356005
P23298	Protein kinase C eta type	Prkch	0.657262069	1.56266634
P23927	Alpha-crystallin B chain	Cryab	0.549626596	1.555245656
P24288	Branched-chain-amino-acid aminotransferase, cytosolic	Bcat1	0.651778664	1.697720252
P25911	Tyrosine-protein kinase Lyn	Lyn	0.79279044	1.442995613
P28798	Progranulin	Grn	0.645125472	1.339155655
P41242	Megakaryocyte-associated tyrosine-protein kinase	Matk	0.568717607	1.70906332
P43883	Perilipin-2	Plin2	0.610554981	1.72825748
P48036	Annexin A5	Anxa5	0.687029392	1.435505226
P49446	Receptor-type tyrosine-protein phosphatase epsilon	Ptpre	0.79828216	1.636672446
P50427	Steryl-sulfatase	Sts	0.695983487	1.526770237
P50543	Protein S100-A11	S100a11	0.682927439	1.511632053
P54763	Ephrin type-B receptor 2	Ephb2	0.665503893	1.284773575
P56375	Acylphosphatase-2	Acyp2	0.589639337	1.344562254
P62983	Ubiquitin-40S ribosomal protein S27a	Rps27a	0.773990343	1.21193522
P68254	14-3-3 protein theta	Ywhaq	0.785198761	1.210454097
P70180	Atrial natriuretic peptide receptor 3	Npr3	0.593193139	1.487836231
P70387	Hereditary hemochromatosis protein homolog	Hfe	0.768285731	1.349846034
P97298	Pigment epithelium-derived factor	Serpinf1	0.747353637	1.305666984
P97467	Peptidyl-glycine alpha-amidating monooxygenase	Pam	0.579600387	1.552475558
Q00612	Glucose-6-phosphate 1-dehydrogenase X	G6pdx	0.653932733	1.300257577
Q00724	Retinol-binding protein 4	Rbp4	0.567152695	1.364799047
Q00941	Granulocyte-macrophage colony-stimulating factor receptor subunit alpha	Csf2ra	0.647337505	1.368877519
Q03350	Thrombospondin-2	Thbs2	0.733103206	1.350827671
Q05793	Basement membrane-specific heparan sulfate proteoglycan core protein	Hspg2	0.666620077	1.26311188
Q3TTY5	Keratin, type II cytoskeletal 2 epidermal	Krt2	0.551401154	1.33451487
Q3UQ28	Peroxidasin homolog	Pxdn	0.560483414	2.667881936
Q3UV17	Keratin, type II cytoskeletal 2 oral	Krt76	0.526516721	1.362560015
Q4ZJN1	Complement C1q and tumor necrosis factor-related protein 9	C1qtnf9	0.703123667	1.397849658
Q562D6	tRNA (adenine(37)-N6)-methyltransferase	Trmo	0.269978943	2.993587642
Q5SXY1	Cytospin-B	Specc1	0.628059005	1.868882268
Q5U4D9	THO complex subunit 6 homolog	Thoc6	0.770677586	1.207621793
Q60675	Laminin subunit alpha-2	Lama2	0.61438	1.520674813
Q60870	Receptor expression-enhancing protein 5	Reep5	0.608420449	1.317769204
Q61699	Heat shock protein 105 kDa	Hsph1	0.798913398	1.462266446
Q62048	Astrocytic phosphoprotein PEA-15	Pea15	0.682596546	1.34870236
Q62261	Spectrin beta chain, non-erythrocytic 1	Sptbn1	0.740102084	1.214602281
Q64449	C-type mannose receptor 2	Mrc2	0.63460548	1.459026095
Q6IRU5	Clathrin light chain B	Cltb	0.762439764	1.217178585
Q6TCG2	Membrane progesterone receptor epsilon	Paqr9	0.620337924	1.985498298
Q71LX4	Talin-2	Tln2	0.721065361	1.205132269
Q80V53	Carbohydrate sulfotransferase 14	Chst14	0.666580674	1.367212788
Q8BG73	SH3 domain-binding glutamic acid-rich-like protein 2	Sh3bgrl2	0.755024856	1.533962261
Q8BGA2	LHFPL tetraspan subfamily member 2 protein	Lhfpl2	0.495844074	1.439941209
Q8BGY9	High affinity choline transporter 1	Slc5a7	0.644645782	1.980018733
Q8BHL5	Engulfment and cell motility protein 2	Elmo2	0.797536093	1.259214907
Q8BHZ0	CYFIP-related Rac1 interactor A	Cyria	0.790382516	1.222435043
Q8BI08	Protein MAL2	Mal2	0.786294133	1.441881996
Q8BPS4	Integral membrane protein GPR180	Gpr180	0.331242807	1.361768073
Q8BZ03	Serine/threonine-protein kinase D2	Prkd2	0.652307623	1.231827765
Q8C142	Low density lipoprotein receptor adapter protein 1	Ldlrap1	0.703602688	1.392901081
Q8C147	Dedicator of cytokinesis protein 8	Dock8	0.789642671	1.517773691
Q8CGA4	Maturin	Mturn	0.491145	1.578948145
Q8CHH9	Septin-8	Septin8	0.686151817	1.372095604
Q8JZK9	Hydroxymethylglutaryl-CoA synthase, cytoplasmic	Hmgcs1	0.641253478	1.406928093
Q8K3K8	Optineurin	Optn	0.680041902	1.365399359
Q8R238	Serine dehydratase-like	Sdsl	0.70692237	1.290868637
Q8VD33	Small glutamine-rich tetratricopeptide repeat-containing protein beta	Sgtb	0.472949058	1.371492625
Q91XD7	Protein disulfide isomerase Creld1	Creld1	0.6104103	1.326438835
Q91YX5	Acyl-CoA:lysophosphatidylglycerol acyltransferase 1	Lpgat1	0.701753748	1.380005096
Q91ZF0	DnaJ homolog subfamily C member 24	Dnajc24	0.731687837	1.305430535
Q921Q3	Chitobiosyldiphosphodolichol beta-mannosyltransferase	Alg1	0.60958506	1.247437542
Q923D4	Splicing factor 3B subunit 5	Sf3b5	0.821544189	1.219278931
Q99J47	Dehydrogenase/reductase SDR family member 7B	Dhrs7b	0.71252128	1.269246863
Q99PJ0	Neurotrimin	Ntm	0.659723271	1.360605816
Q9CW42	Mitochondrial amidoxime-reducing component 1	Mtarc1	0.547778041	1.312522546
Q9CWS0	N(G),N(G)-dimethylarginine dimethylaminohydrolase 1	Ddah1	0.38989088	2.640649238
Q9CXT7	Transmembrane protein 192	Tmem192	0.811599619	1.447067984
Q9CY64	Biliverdin reductase A	Blvra	0.760695509	1.40052152
Q9D379	Epoxide hydrolase 1	Ephx1	0.700642139	1.425222882
Q9D3P8	Plasminogen receptor (KT)	Plgrkt	0.660636171	1.344838373
Q9D7G0	Ribose-phosphate pyrophosphokinase 1	Prps1	0.708687594	1.652020885
Q9EPL9	Peroxisomal acyl-coenzyme A oxidase 3	Acox3	0.774813018	1.314661776
Q9ES57	Cell surface glycoprotein CD200 receptor 1	Cd200r1	0.477497904	1.726018437
Q9JKX3	Transferrin receptor protein 2	Tfr2	0.441377047	2.209652728
Q9JL62	Glycolipid transfer protein	Gltp	0.732512513	1.432249415
Q9QYR6	Microtubule-associated protein 1A	Map1a	0.567535959	1.750284473
Q9R001	A disintegrin and metalloproteinase with thrombospondin motifs 5	Adamts5	0.635456846	1.324770169
Q9R013	Cathepsin F	Ctsf	0.613397598	1.407441745
Q9Z0F7	Gamma-synuclein	Sncg	0.654712098	1.337344502
Q9Z0J0	NPC intracellular cholesterol transporter 2	Npc2	0.549065572	1.397981367
Q9Z0Z4	Hephaestin	Heph	0.72666994	1.281808528
Q9Z138	Tyrosine-protein kinase transmembrane receptor ROR2	Ror2	0.628599362	1.370936865

### 3.5 Functional classification of DEPs

**Figure 5:**
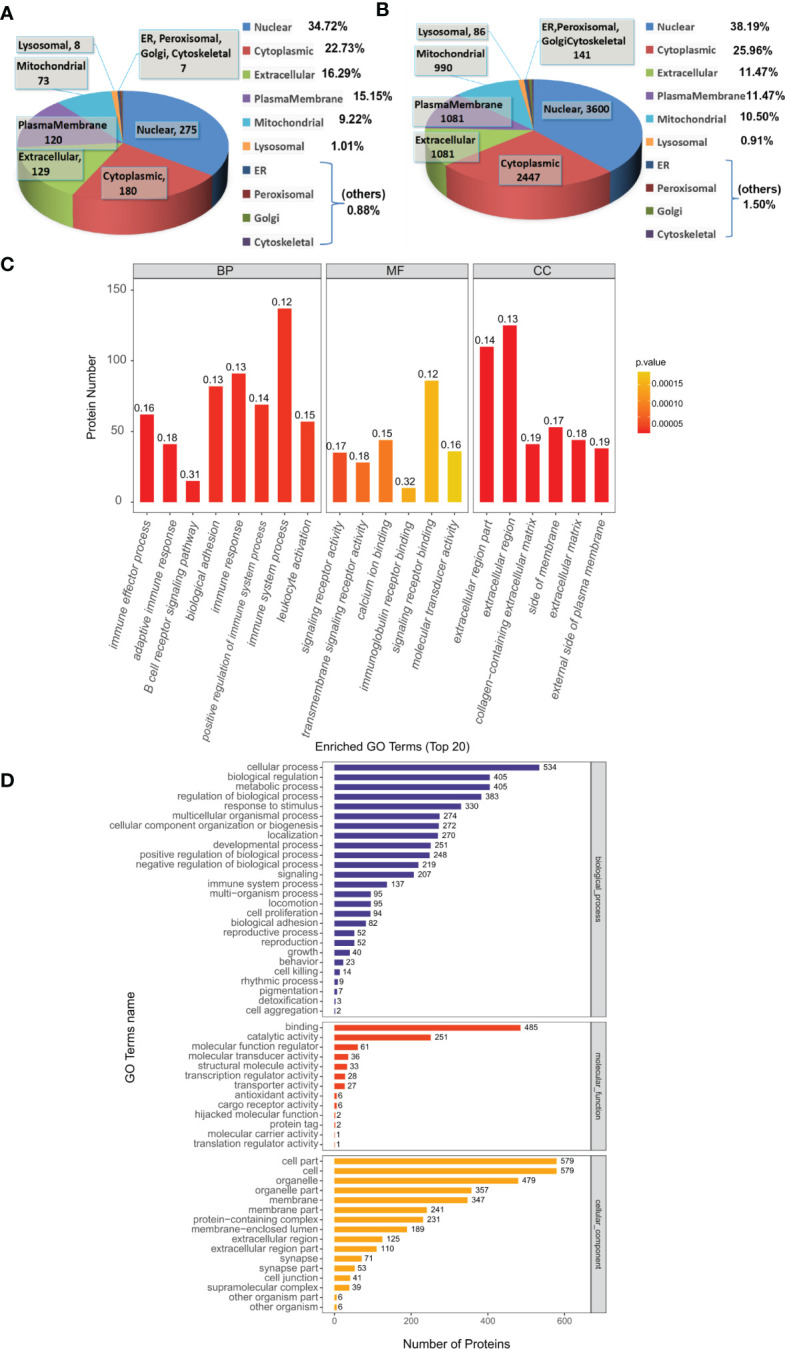
Functional analysis of DEPs. **(A, B)** Subcellular localization analysis of DEPs in Sema/HFD group and total proteome. The numbers show the proteins located in different subcellular components. **(C)** Overall enriched GO terms of DEPs between the Sema and HFD groups. BP: biological processes; MF: molecular functions; CC: cellular components. **(D)** Top 20 enriched GO analyses of DEPs. The color gradient from orange to red represents the p-value. The numbers above the bar charts represent the rich factor (rich factor ≤ 1).

We used the subcellular structure prediction software CELLO to analyze the subcellular location of all the DEPs. DEPs were mainly located in nuclear (34.72% in Sema/HFD, 38.19% in total). The remaining DEPs were mainly located in cytoplasmic (22.73% in Sema/HFD, 25.96% in total), extracellular (16.29% in Sema/HFD, 11.47% in total), plasma membrane (15.15% in Sema/HFD, 11.47% in total) and mitochondrial (9.22% in Sema/HFD, 10.5% in total; **Figures 5A, B**).

The DEPs were then analyzed against the GO database using three sets of ontologies: biological process (BP), molecular function (MF), and cellular component (CC). In the Sema/HFD group, the most enriched GO terms of BP, MF, and CC were annotated as immune effector process, signaling receptor activity, and extracellular region part, respectively (**Figures 5C, D**, **Table 3**). Other important BPs were included in adaptive immune response, B cell receptor signaling pathway, biological adhesion, immune response, positive regulation of immune system process and immune system process, and leukocyte activation (**Figure 5C**). Other important MFs included transmembrane signaling receptor activity, calcium ion binding, immunoglobulin receptor binding, and signaling receptor binding molecular transducer activity (**Figure 5C**). Other important CCs included extracellular region, collagen-containing extracellular matrix, side of membrane, extracellular matrix, and external side of the plasma membrane (**Figure 5C**).

**Table 3:** Distribution of proteins and signaling pathways between Sema and HFD groups, based on GO and KEGG analysis.

Terms	Count	*p* Value	FDR	richFactor	Accession
GO (gene ontology)					
immune effector process (BP)	62	7.62E-07	0.001277484	0.15736041	P08508,P15702,P08101,P06339,P04104,Q3SXB8,P00493,P97821,P01899,P36371,P70387,O55186,Q3TBT3,P28798,Q9ESD7,Q8BVZ5,P13597,P24063,Q8CIH5,P16045,Q8K394,P01872,P35762,P25911,P97313,Q9EQU3,Q9D8I1,Q8BMI0,Q8HWB0,P01837,P18528,Q61475,P01844,P18526,P18527,P06327,P01821,P25446,Q9JIA7,Q3U1T9,Q8R5F7,Q99J87,P21958,P22682,Q05144,P14234,Q6BCL1,Q9ERI2,Q9EP53,P10107,P42232,Q8BGF6,Q00724,Q60710,Q9JL16,Q9Z2F2, P58681,Q8VI93,Q9D483,Q99P72,Q08857,Q8K3H0
signaling receptor activity (MF)	35	3.11E-05	0.011700757	0.17241379	Q9JIA7,Q62312,P54763,Q9Z138,P01872,P15702,Q9EQU3,P58681,Q704Y3,P20352,Q00941,P27931,P10493,O88495,Q8BUD0,P51675,A2A8L5,Q8BPB5,O35607,P25446,P70180,P06332,P70206,P70207,P08101,P08508,P24063,P98156,Q08857,P09055,P04925,O70309,Q64449,Q9ES57,Q6TCG2
extracellular region part (CC)	110	2.39E-08	0.000108474	0.13977128	P02469,P10107,P05064,P48036,P01872,P13020,P16045,Q8R2Y2,Q05816,O35206,O09164,P01899,Q8BPB5,Q3UQ28,P07309,P01631,Q99JR5,O08692,P21956,Q9Z0J0,Q9R013,Q640N1,P97298,O35074,Q62426,P51437,Q64191,Q08879,P06339,P97821,P28798,Q9QZF2,P70387,Q00724,P25446,P20352,Q4ZJN1,P98156,P01636,P11152,P13597,P30681,P10810,Q8VCI0,Q9Z1P8,P01630,P15702,P61148,P70663,P27005,P04940,P97467,Q9R001,P28301,P01723,P47931,Q8HWB0,Q9R1B9,Q8BVZ5,P41245,P12388,O88839,O35684,P01674,Q9CZJ1,P06802,O35930,Q8CC36,Q3SXB8,A2AJ76,P01634,Q99PJ0,Q9Z0E6,Q61107,Q05793,P02468,P10493,O88322,Q03350,Q80WM5,P01837,P18528,P01844,P18526,P18527,P06327,P01821,P10649,P42208,Q9D6Y7,Q9CQ48,P48725,Q8BGA5,Q61292,P97927,P14824,Q60675,P04104,P14069,P50543,P70207,Q61878,Q99KG5,P09055,O09131,Q3V1T4,O35566,Q9CQF9,P35762,Q91ZR2
Kyoto Encyclopedia of Genes and genomes (KEGG) pathways					
Valine, leucine and isoleucine biosynthesis	3	0.000596964	0.172522484	1	O35855 Q8R238 P24288
					
ECM-receptor interaction	11	0.001215489	0.175638137	0.23913044	Q05793 Q61292 P97927 P02469 Q60675 P09055 Q08857 P43406 O70309 Q03350 O35930
African trypanosomiasis	6	0.001934429	0.186350014	0.35294118	P97927 P21279 P25446 P13597 Q9EQU3 A3KGF7
Fluid shear stress and atherosclerosis	16	0.003560664	0.205806368	0.17582418	P10649 P49817 Q9WVC3 O09131 P43406 Q05144 Q9QZF2 P24472 P13597 P31750 P27931 O35607 Q9Z2X8 P63166 P41245 O35904
Bacterial invasion of epithelial cells	12	0.003255416	0.205806368	0.20338983	P09055 P49817 Q9WVC3 Q8CHH9 P42208 Q8BZ98 Q8BHL5 Q6IRU5 P22682 Q61140 Q9QYY0 O35904
Human cytomegalovirus infection	20	0.004976538	0.210314901	0.15625	P01899 P36371 P21279 P43406 P21958 Q05144 P06339 Q9R233 P31938 Q921J2 P25446 Q61140 P31750 P61953 P51675 Q3TBT3 P97490 O35904 A3KGF7 Q9EP53
Hematopoietic cell lineage	8	0.005094133	0.210314901	0.24242424	Q08857 O55186 Q61475 P10810 P27931 Q00941 P06332 O35930
Focal adhesion	18	0.016887784	0.287092327	0.144	Q71LX4 Q61292 P97927 P02469 P57780 Q60675 P09055 P49817 Q9WVC3 P43406 Q05144 P31938 O70309 Q61140 Q03350 P31750 P70182 O35904
Fc gamma R-mediated phagocytosis	12	0.016378703	0.287092327	0.16666667	P13020 P25911 Q8CIH5 Q05144 P08101 P31938 P28667 P31750 Q7TQF7 Q9JIA7 P70182 O35904
Cell adhesion molecules	10	0.010431386	0.287092327	0.19230769	P09055 P01899 P43406 P06339 A2A8L5 P13597 P15702 P06332 Q9Z261 P27573

### 3.6 KEGG pathways

**Figure 6:**
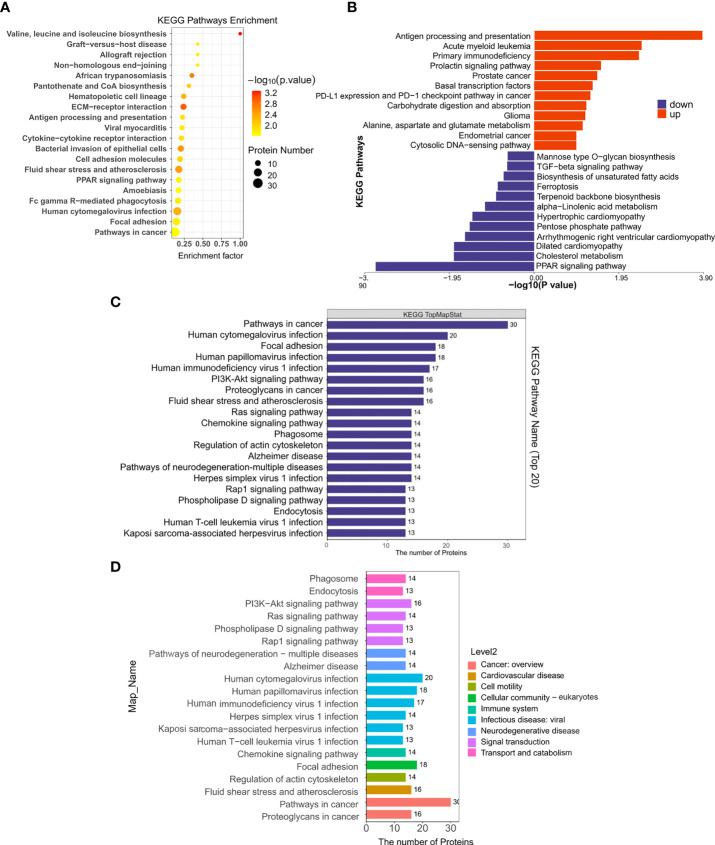
The KEGG pathway enrichment of DEPs. **(A)** KEGG pathway enrichment bubble chart. The horizontal axes represent the enrichment factor (enrichment factor ≤ 1). The vertical axis represents the statistical results of DEPs under the Top 20 KEGG pathways. The color gradient from green to red represents the p-value; the closer to the red color, the lower the p-value and the higher the significance level corresponding to the enrichment. **(B)** Significantly enriched pathways in up/down-regulated DEPs in the Sema/HFD group. **(C)** Numbers of DEPs under Top 20 KEGG pathways. **(D)** Numbers of DEPs under level 2 KEGG pathways.

By searching the major biological pathways and relevant regulatory processes involved in the KEGG, we analyzed all of the DEPs in the Sema and HFD groups. Valine, leucine, and isoleucine biosynthesis, ECM-receptor interaction, African trypanosomiasis, fluid shear stress and atherosclerosis, bacterial invasion of epithelial cells, human cytomegalovirus infection, hematopoietic cell lineage, focal adhesion, Fc gamma R-mediated phagocytosis, and cell adhesion molecules, resulted as significant enrichment pathways (**Figure 6A**, **Table 3**). Most of the DEPs were enriched in pathways related to cancer and human cytomegalovirus infection (**Figures 6C, D**).

**Figure 7:**
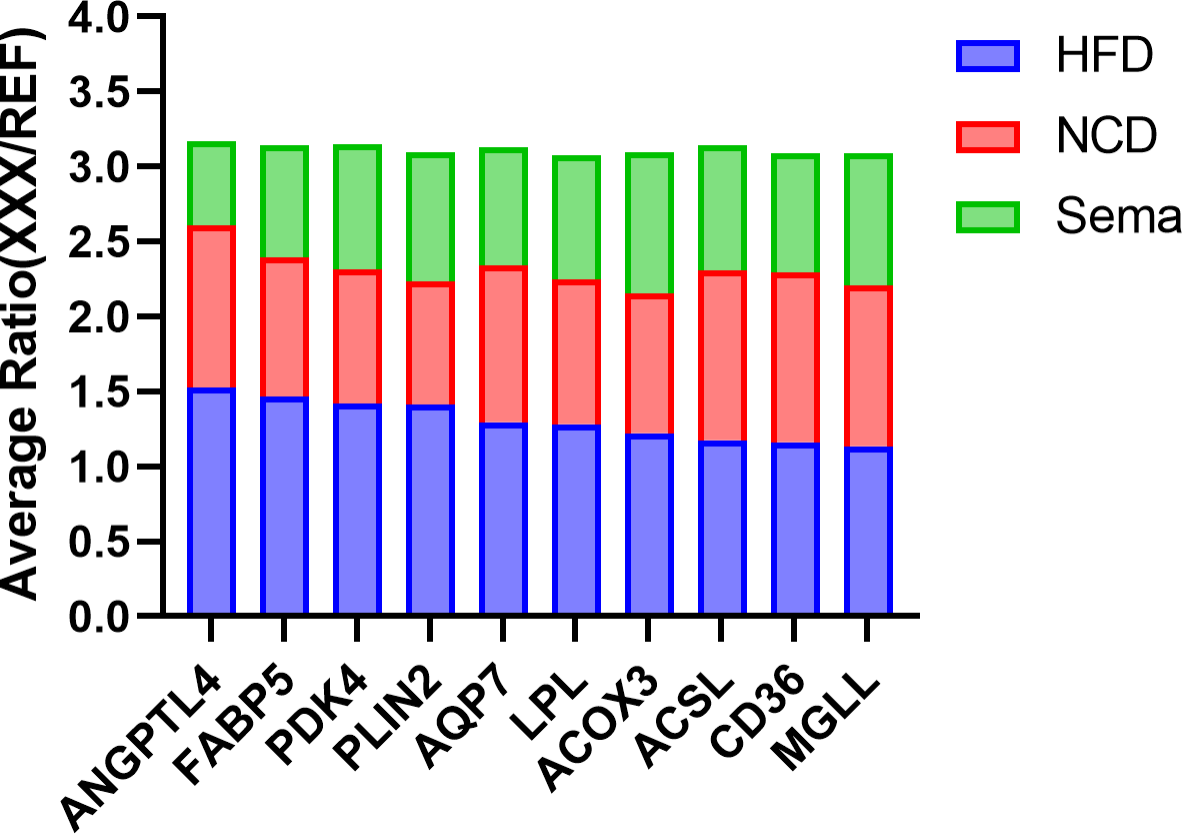
The Average Ratio (XXX/REF) of lipid metabolism proteins in HFD, NCD, and Sema groups. Mean relative protein expression of the sample group relative to the internal reference (REF).

Moreover, up-regulated DEPs were found to be enriched in antigen processing and presentation, human immunodeficiency virus 1 infection, herpes simplex virus 1 infection, acute myeloid leukemia, etc. (**Figure 6B**, **Table 4**). Down-regulated DEPs were found to be enriched in the PPAR signaling pathway and cholesterol metabolism (**Figure 6B**, **Table 4**). Interestingly, we found 10 proteins involved in the fatty acid uptake, lipid storage, unsaturated fatty acid synthesis, lipid peroxidation, and glycerol efflux down-regulated in Sema/HFD group, including CD36 FABP5, ACSL, ACOX3, PLIN2, ANGPTL4, LPL, MGLL, AQP7, and PDK4 (**Figure 7**, **Table 5**). In addition, the expression of PDK4, ACOX3, PLIN2 were up-regulated with HFD and significantly reversed by Sema. Taken together, these findings suggested that semaglutide treatment might have beneficial effects on adipose tissues through the regulation of lipid uptake, lipid storage, and lipolysis in white adipose tissue (**Figures S3**–**6**).

**Table 4:** Significantly enriched pathways in up/down regulated DEPs between Sema and HFD groups identified through KEGG pathway enrichment analysis.

Terms	Count	*p* Value	FDR	richFactor	Accession	Sema/HFD DEPs Regulated Type
Antigen processing and presentation	7	0.00013769	0.033596295	0.22580645	P01899 P97372 P36371 P21958 P06339 Q9R233 P06332	up
Human immunodeficiency virus 1 infection	14	0.000609053	0.054097938	0.10447761	P01899 Q60710 P36371 P21958 Q8CIH5 Q05144 P06339 Q9R233 P31938 P31750 Q3TBT3 Q9DB50 P06332 O35904	up
Herpes simplex virus 1 infection	12	0.000665139	0.054097938	0.11428571	P01899 P36371 P21958 P06339 Q9R233 Q8R5F7 P31750 Q3TBT3 Q9EQU3 Q8VI93 O35904 Q9EP53	up
Human cytomegalovirus infection	13	0.001234466	0.075302447	0.1015625	P01899 P36371 P21958 Q05144 P06339 Q9R233 P31938 P31750 P51675 Q3TBT3 O35904 A3KGF7 Q9EP53	up
Acute myeloid leukemia	6	0.003441545	0.13284056	0.15384615	P42232 P31938 P10810 P31750 P17433 O35904	up
Cell adhesion molecules	7	0.003512205	0.13284056	0.13461539	P01899 P06339 P13597 P15702 P06332 Q9Z261 P27573	up
Primary immunodeficiency	3	0.003983193	0.13284056	0.33333333	P36371 P21958 P06332	up
Natural killer cell mediated cytotoxicity	7	0.004355428	0.13284056	0.12962963	P01899 Q8CIH5 Q05144 P06339 P31938 P13597 O35904	up
Human T-cell leukemia virus 1 infection	10	0.005680549	0.150034667	0.09803922	P01899 P42232 P06339 P31938 P13597 P31750 P27931 P17433 P06332 O35904	up
Fc gamma R-mediated phagocytosis	8	0.006148962	0.150034667	0.11111111	Q8CIH5 Q05144 P08101 P31938 P28667 P31750 Q7TQF7 O35904	up
ECM-receptor interaction	10	3.35E-05	0.008284126	0.2173913	Q05793 Q61292 P97927 P02469 Q60675 P09055 Q08857 P43406 O70309 Q03350	down
PPAR signaling pathway	10	0.000226586	0.025015808	0.1754386	P41216 Q62417 Q05816 Q08857 P43883 Q9EPL9 Q8JZK9 P11152 Q9Z1P8 O54794	down
Bacterial invasion of epithelial cells	10	0.000303836	0.025015808	0.16949153	P09055 P49817 Q9WVC3 Q8CHH9 P42208 Q8BZ98 Q8BHL5 Q6IRU5 Q61140 Q9QYY0	down
Focal adhesion	14	0.001675795	0.103480359	0.112	Q71LX4 Q61292 P97927 P02469 P57780 Q60675 P09055 P49817 Q9WVC3 P43406 O70309 Q61140 Q03350 P70182	down
Valine, leucine and isoleucine biosynthesis	2	0.006098131	0.301247675	0.66666667	Q8R238 P24288	down
Small cell lung cancer	7	0.011087266	0.44441595	0.12962963	Q61292 P97927 P02469 Q60675 P09055 P43406 O89106	down
Cholesterol metabolism	5	0.014282383	0.44441595	0.15625	Q08857 Q9Z0J0 P11152 Q9Z1P8 Q8C142	down
Dilated cardiomyopathy	6	0.014394039	0.44441595	0.13636364	P48678 Q60675 P09055 P43406 O70309 P97490	down
Arrhythmogenic right ventricular cardiomyopathy	5	0.025679591	0.704762112	0.13513514	P48678 Q60675 P09055 P43406 O70309	down
Pentose phosphate pathway	4	0.033096704	0.8174886	0.14814815	P05064 Q00612 Q9D7G0 Q8R1Q9	down

**Table 5:** DEPs down -regulated in the Sema/HFD group modulating the lipid metabolism.

Accession	Protein Name	Gene Name	Sema / HFD
P41216	Long-chain-fatty-acid--CoA ligase 1	Acsl1	0.714539589
Q05816	Fatty acid-binding protein 5	Fabp5	0.513802071
O54794	Aquaporin-7	Aqp7	0.6124858
P43883	Perilipin-2	Plin2	0.610554981
O35678	Monoglyceride lipase	Mgll	0.775676329
Q9EPL9	Peroxisomal acyl-coenzyme A oxidase 3	Acox3	0.774813018
Q9Z1P8	Angiopoietin-related protein 4	Angptl4	0.366305511
P11152	Lipoprotein lipase	Lpl	0.649312671
Q08857	Platelet glycoprotein 4	Cd36	0.691987249
O70571	[Pyruvate dehydrogenase (acetyl-transferring)] kinase isozyme 4, mitochondrial	Pdk4	0.5890158

## 4 Discussion

Obesity is a highly prevalent, chronic, relapsing disease requiring long-term management (19–21). Semaglutide is a potent long-acting glucagon-like peptide-1 (GLP-1) analogue that requires once-weekly administration (22). It regulates blood glucose *via* the incretin pathway, stimulating insulin and inhibiting glucagon secretion in a glucose-dependent manner, leading to lower blood glucose levels with low risk for hypoglycaemia (23). Semaglutide provides weight-loss effects, and greater reduction in fat mass than that reported with liraglutide (3). The Semaglutide Treatment Effect in People with obesity (STEP) clinical trial programme provides evidences that improvements in cardiometabolic risk factors, including high blood pressure, atherogenic lipids and benefits on physical function and quality of life were seen with semaglutide 2.4 mg (24).

To determine the role of semaglutide in obese mice, we performed intraperitoneally injections of semaglutide to HFD-induced obese mice, whereas the NCD and HFD groups were treated with saline for 12 weeks. As expected, we observed that semaglutide exerted a weight-loss effect by lowering the proportion of visceral fat mass and increasing the brown fat mass relative to total body mass. Both plasma lipids (cholesterol and triglycerides) and plasma lipoprotein (LDL) levels were significantly decreased after semaglutide treatment. Semaglutide significantly reduced blood glucose concentrations in glucose tolerance tests. However, plasma levels of fasting insulin decreased significantly in the semaglutide treated mice, when it is known that semaglutide does the opposite in humans (25). A potential factor explaining this observation may be the loss of body weight during the course of the experiment, possibly impacting insulin resistances. A large board of studies and clinical correlations has suggested that hyperinsulinemia is associated with obesity, and there is a close relationship between hyperinsulinemia and dyslipidemia (26, 27). Recent *in vivo* evidence has also shown that reducing circulating insulin levels may protect and reverse adiposity, insulin resistance, and hyperglycemia that is associated with obesity (28). A previous study discovered that plasma fasting insulin and leptin levels decreased in the GLP-1 (rhGLP-1) Beinaglutide (BN) treated mice, suggesting that BN could reduce hyperinsulinemia and hyperleptinemia associated with obesity (29). Based on the above findings, in this study we indicate that semaglutide could reduce hyperinsulinemia and promote the insulin sensitivity in obese mice.

Adipose tissues are important regulators of whole-body energy homeostasis. In this study, we focused on the role of semaglutide on adipose metabolism and found that it has several beneficial effects on the adipose tissues of obese mice.

First, cell size and turnover of adipocytes are major determinants of fat tissue metabolism and mass, the alterations of which are associated with pathological conditions (16). Semaglutide-treated mice not only show dramatically reduced adipose tissue weight, H&E staining also showed that the large lipid droplets with distinct morphologies in the adipocytes of obese mice were alleviated by semaglutide. Hypertrophy, an increase in cell size of adipocyte is closely associated with dyslipidemia and insulin resistance in humans (30, 31). A reduction in the diameter of adipocytes induced by semaglutide may indicate improvement of insulin sensitivity in the fat tissues of obese mice.

Second, although the changes of adipose tissue respond to GLP-1 analogues treatment has been studied from the gene expression, lipidomic perspectives (29, 32),comprehensive protein level profiling is rare. Gene expression and protein expression diverge (33). Profiling the adipose tissue proteomic signatures identified pathways hat were not available through gene expression studies (34).

To provide greater insight into the role of semaglutide in obese white adipose tissue, we applied TMT proteomic approach to analyze a total of 7553 proteins quantifiable in the three experimental groups. There were 683 DEPs in the HFD/NCD group and 640 DEPs in the HFD/Sema group, with a total of 141 significant overlapping DEPs. Previous study provided evidence for the presence of GLP-1 receptor in adipose tissue and show that its mRNA and protein expressions increased in visceral adipose depots from morbidly obese patients with a high degree of Insulin resistances (IR) (7). Interestingly, protein O35659 was not identified by TMT LC-MS in our study, which was consistent with another study of h rhGLP-1 Beinaglutide (BN) on adipose tissue (29). Potential explanations of this observation may be that the fat loss effect by semaglutide during the course of the experiment, might lower the degree of IR, leading to a lower expression of Glpr in mice, or the beneficial effects of GLP-1 are associated with the activation of not only the canonical GLP-1 receptor but also an additional, as yet unknown, receptor (8), the effects of semaglutide on adipose tissues might not be through its regulation on expression of GLP-1R. This phenomenon may also be due to technical limitations that the protein extraction does not efficiently extract membrane proteins, the hydrophobic properties of these proteins make full structural and functional characterization challenging because of the need to use detergents or other solubilizing agents when extracting them from their native lipid membranes (35).

It is well established that GLP-1/GLP-1R functions through activation of down-stream PKA-, MAPK-, or AMPK-related signaling pathways in various cells. Xu et al. investigated the contribution of brown remodelling of WAT to the weight-lowering-effect induced by the GLP-1R agonist exenatide, and researched the role of SIRT1 in this process (32). Their findings indicated that exenatide induces the phosphorylation of AMPK which, in turn, activates SIRT1 by regulating NAD+ concentration, triggering a lipolytic cycle. Our data have also shown that semaglutide stimulates the expression of Q8C078 (Camkk2) up-regulated in the obese adipose tissue (**Table S3**). AMPKs can be activated by a Ca2+-dependent pathway mediated by CaMKKb (36, 37). It was indicated that the actions of GLP-1 on VAT should be modulated by sympathetic tone to activate AMPK, leading to decreased lipogenesis and reduced triglyceride content (38). We indicate that semaglutide can activate cAMP-associated signaling. The KEGG analyses of the proteomic data have also shown that PI3K-AKT and PPAR signaling pathways and several lipid-metabolism-related pathways are among the top enriched pathways in the obese adipose tissue with semaglutide treatment.

Notably, the KEGG analyses have shown that down-regulated DEPs in Sema/HFD group were found to be enriched in the cholesterol metabolism and PPAR signaling pathways. These results suggest that semaglutide down-regulates several lipid-metabolism-related proteins in adipose tissues, it might be reducing PPAR pathway activity when PPAR gamma (39) is good for metabolic health. A potential explanation of this observation may be that semaglutide could also be acting on other signals resulted in reducing PPAR activity in the white adipose tissue, but is associated with reducing visceral fat accumulation and plasma lipid level and increasing insulin sensitivity. Shao et al. found that with liraglutide treatment, the lipogenetic transcription factors PPARγ and C/EBPα expressions were both reduced with AMPK activation and Akt suppression in visceral adipose tissue (VAT), which was associated with reduction of lipogenetic process in VAT (10). A recent study also found that GLP‐1 significantly decreased the expression levels of two key markers of adipocyte differentiation, PPARγ and FABP4, as well as increased that of adiponectin in VAT explants from morbidly obese patients, compared with untreated controls (8). This can be interpreted as the reduction of VAT after weight loss with semaglutide treatment might be related to the adipogenesis decreases and lipolysis increases to avoid more fat accumulation and improve metabolic health, which deserves further investigation.

Based on the results of the study, we found 10 proteins significantly concentrated in lipid metabolism down-regulated in Sema/HFD group, including fatty acid transport and oxidation (CD36 FABP5, ACSL, ACOX3). CD36 is a multifunctional glycoprotein that has a critical role in LCFA uptake and transport in adipocytes (40–42). A previous study indicated that during fat depot expansion, CD36 deficiency negatively affects preadipocyte recruitment in mature adipocytes (43). Fatty acid-binding protein 5 (FABP5) delivers specific fatty acids from the cytosol to the nucleus, wherein they activate nuclear receptors (44) and modulate inflammation by regulating PTGES induction *via* NF-kappa-B activation. Long-chain-fatty-acid–CoA ligase 1 (ACSL) plays important roles in lipid metabolism for accelerating fatty acid biosynthesis by both synthesis of cellular lipids, and degradation *via* beta-oxidation (45). Peroxisomal acyl-coenzyme A oxidase 3 (ACOX3) positively regulates fatty acid oxidation (46).

Besides, LPL, PLIN2, ANGPTL4 which positively regulate the process of preadipocyte differentiation (46–48), involved in the adipocyte hypertrophy. Hypertrophic adipocytes are associated with increased insulin resistance (49–51). And MGLL, AQP7, PDK4 all positively regulate energy use in maintaining metabolic balance between carbohydrates and lipids (52–54). All of these 10 proteins had significantly higher expression in the HFD group than in the Sema group and NCD group. In addition, the expression of several proteins was significantly lower in Sema group than that in NCD group (**Figure 7**). It indicates that sema might alleviate the HFD-induced adipose tissue expansion by down-regulated proteins related to the lipid metabolism, both biosynthesis and degradation.

Conclusion: we indicate that semaglutide treatment might have beneficial effects on adipose tissues through the regulation of lipid uptake, lipid storage, and lipolysis in white adipose tissue.

## 5 Conclusion

In the present study, we used a quantitative proteomics approach to investigate alterations of eWAT protein expression in obese mice and examined the therapeutic effect of semaglutide on peripheral metabolic health. Our results suggested that semaglutide could remarkably improve glucose and lipid metabolism, and we indicate that semaglutide treatment might have beneficial effects on adipose tissues through the regulation of lipid uptake, lipid storage, and lipolysis in white adipose tissue.

## Data availability statement

The original contributions presented in the study are included in the article/**Supplementary Material**. Further inquiries can be directed to the corresponding author.

## Ethics statement

The animal study was reviewed and approved by Hebei General Hospital institutional animal care and conducted according to the AAALAC and the IACUC guidelines.

## Author contributions

RZ and SC carried out the studies, participated in collecting data, and drafted the manuscript. RZ and SC performed the statistical analysis and participated in its design. RZ and SC participated in the acquisition, analysis, or interpretation of data and drafting of the manuscript. All authors contributed to the article and approved the submitted version.
