# Supplementary figures and images for: Proteomic analysis reveals semaglutide impacts lipogenic protein expression in epididymal adipose tissue of obese mice
Source: Front Endocrinol (Lausanne). 2023 Mar 21;14:1095432. doi: 10.3389/fendo.2023.1095432 (PMC10070826; doi:10.3389/fendo.2023.1095432)

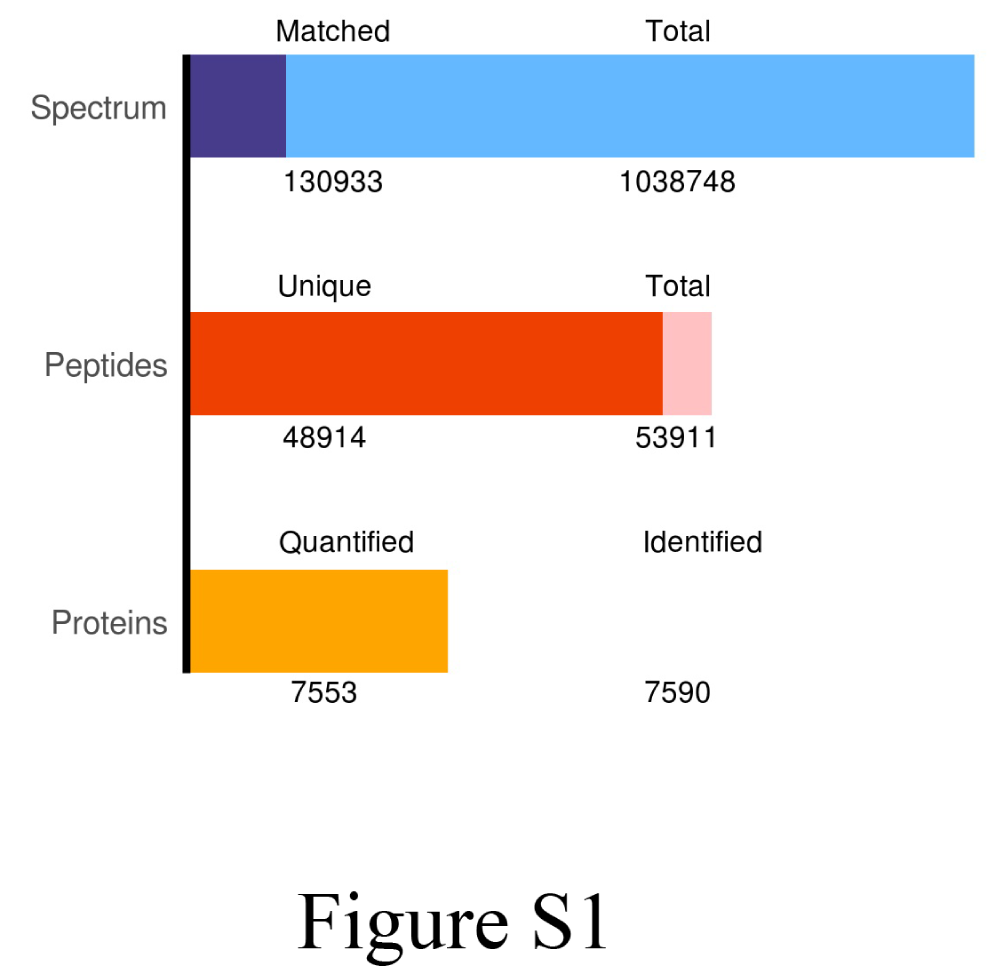

Supplement: Supplementary file 1 [file Image_1.tif]

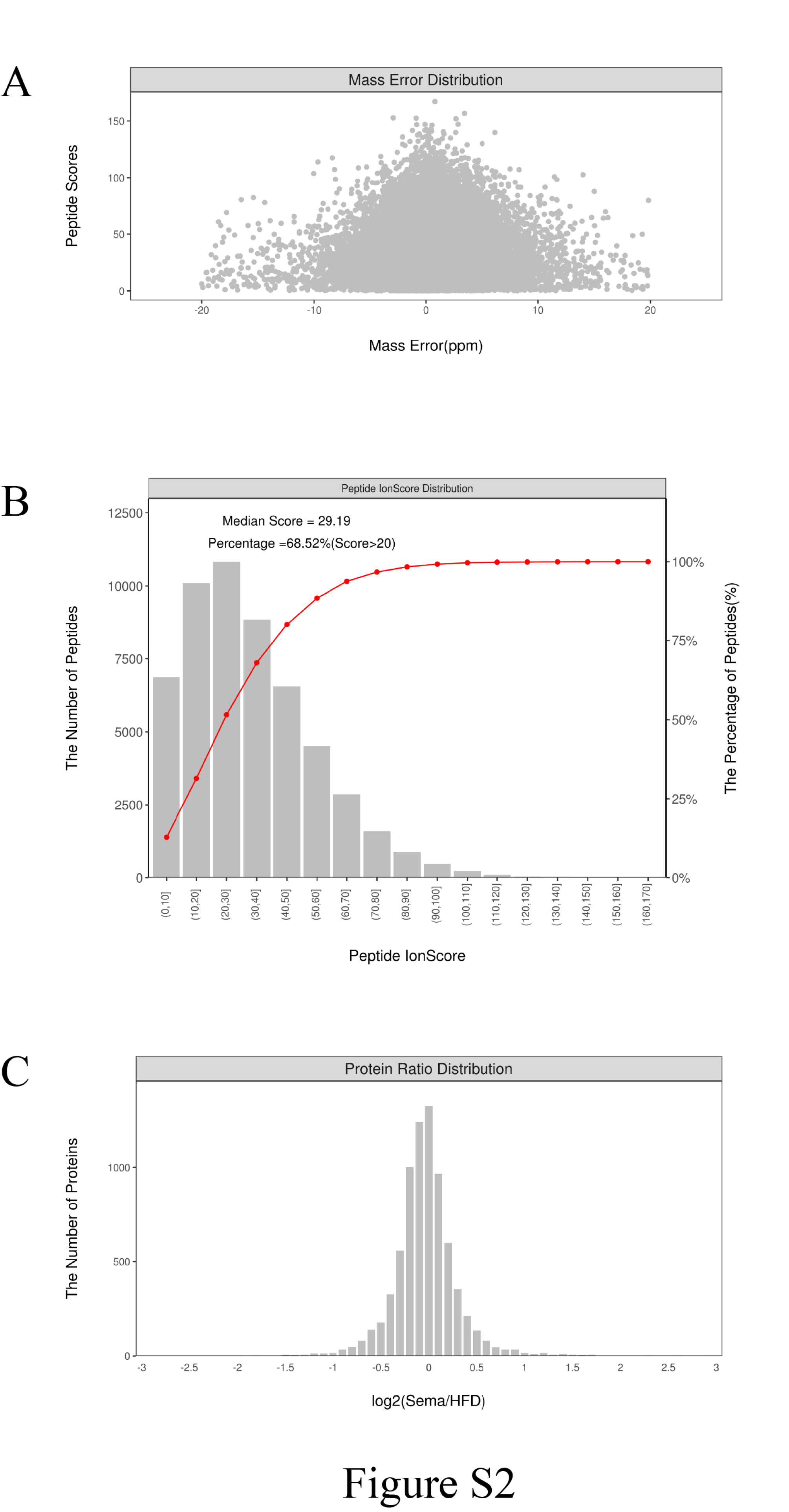

Supplement: Supplementary file 2 [file Image_2.tif]

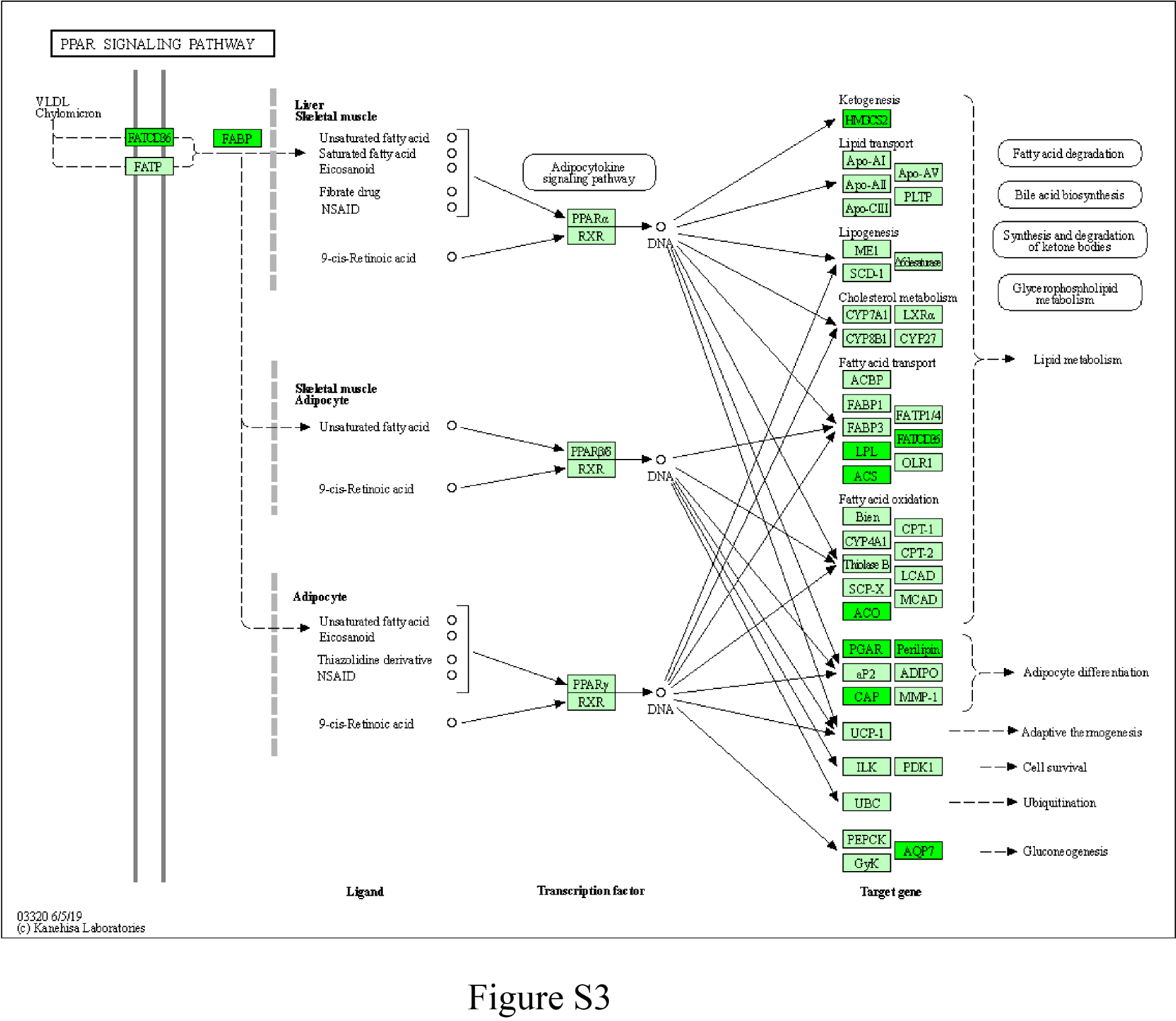

Supplement: Supplementary file 3 [file Image_3.tif]

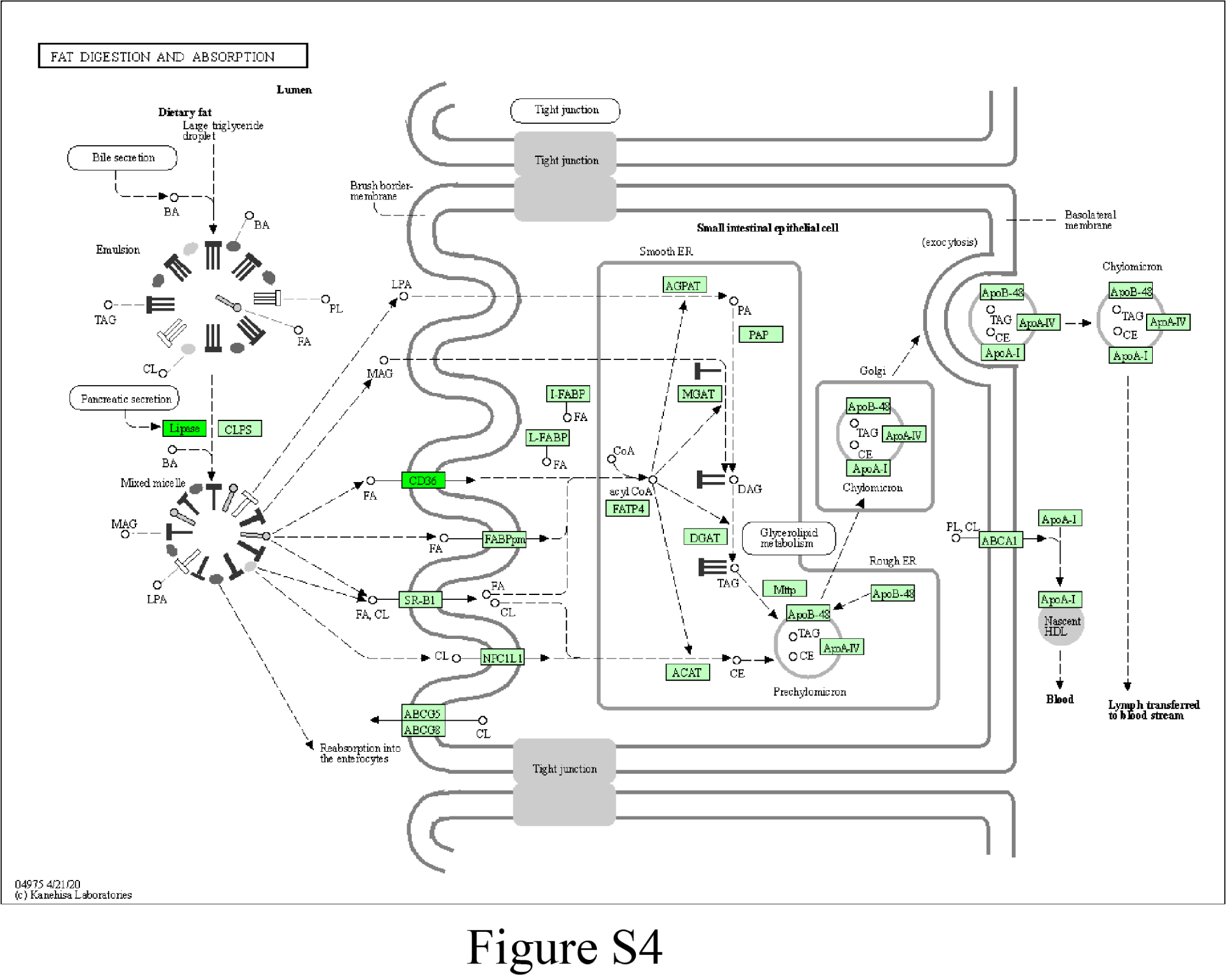

Supplement: Supplementary file 4 [file Image_4.tif]

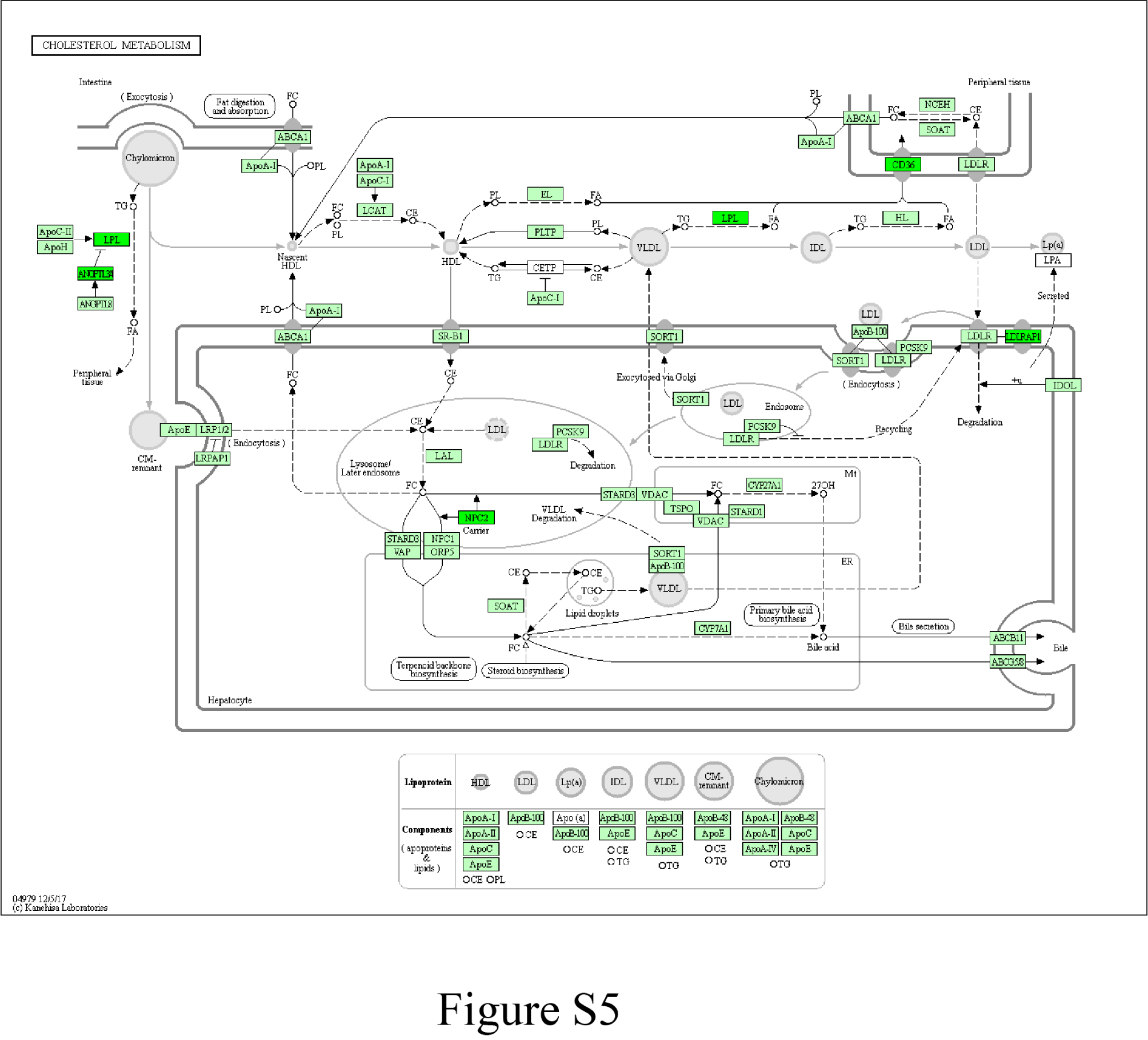

Supplement: Supplementary file 5 [file Image_5.tif]

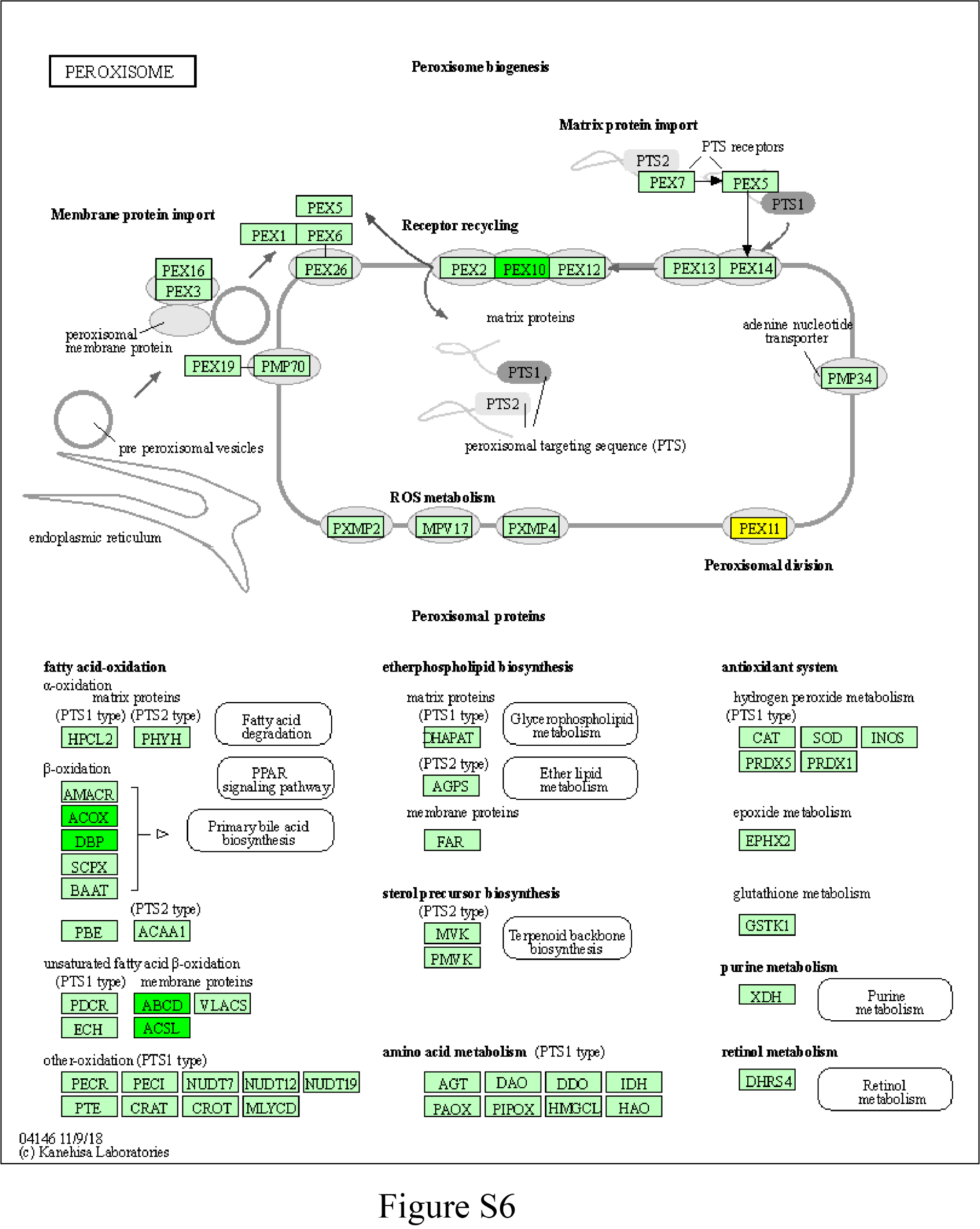

Supplement: Supplementary file 6 [file Image_6.tif]
