# Supplementary material for: Proteomic analysis reveals semaglutide impacts lipogenic protein expression in epididymal adipose tissue of obese mice
Source: Front Endocrinol (Lausanne). 2023 Mar 21;14:1095432. doi: 10.3389/fendo.2023.1095432 (PMC10070826; doi:10.3389/fendo.2023.1095432)
Supplement: Supplementary file 7 [file Table_1.docx]

Table S1 Parameters and instructions of MASCOT engine search

| Item | Value |
| --- | --- |
| Enzyme | Trypsin |
| Max Missed Cleavages | 2 |
| Fixed modifications | Carbamidomethyl (C),  iTRAQ 4/8plex (N-term), iTRAQ 4/8plex (K)  TMT 6/10/16 plex (N-term), TMT 6/10/16 plex (K) |
| Variable modifications | Oxidation (M) , iTRAQ 4/8plex (Y), TMT 6/10/16plex (Y) |
| Peptide Mass Tolerance | ± 20 ppm |
| Fragment Mass Tolerance | 0.1Da |
| Database | See the project report |
| Database pattern | Decoy |
| Peptide FDR | ≤0.01 |
| Protein Quantification | The protein ratios are calculated as the median of only unique peptides of the protein |
| Experimental Bias | Normalizes all peptide ratios by the median protein ratio. The median protein ratio should be 1 after the normalization. |
